# Molecular Mechanisms and Therapeutic Targets of RNA-Based and Traditional Lipid-Lowering Agents in Residual Cardiovascular Risk: A Scoping Review of Key Directions Towards Future Perspectives

**DOI:** 10.3390/biom16060807

**Published:** 2026-05-29

**Authors:** Diana Tatarciuc, Irina Mihaela Esanu, Mioara Florentina Trandafirescu, Ana Maria Raluca Pauna, Teodor Flaviu Vasilcu, Iolanda Foia, Adina Oana Armencia, Magda Ecaterina Antohe, Dragos Catalin Ghica, Ovidiu Stamatin, Roxana Ionela Vasluianu

**Affiliations:** Faculty of Medicine, Grigore T. Popa University of Medicine and Pharmacy, 700115 Iasi, Romania; diana.tatarciuc@umfiasi.ro (D.T.); irina.esanu@umfiasi.ro (I.M.E.); mio.trandafirescu@umfiasi.ro (M.F.T.); paunaanamariaraluca@gmail.com (A.M.R.P.); teodor-flaviu.tsr.vasilcu@umfiasi.ro (T.F.V.); iolanda.foia@umfiasi.ro (I.F.); magda.antohe@umfiasi.ro (M.E.A.); dragos.ghica@yahoo.ro (D.C.G.); ovidiu.stamatin@umfiasi.ro (O.S.); roxana.vasluianu@umfiasi.ro (R.I.V.)

**Keywords:** RNA interference, small interfering RNA, antisense oligonucleotide, gene silencing, RISC complex, RNase H, GalNAc conjugation, lipoprotein(a), *PCSK9*, *APOC3*, *ANGPTL3*, residual cardiovascular risk, dyslipidemia

## Abstract

Residual cardiovascular risk arises from dysregulated expression of genes encoding apolipoprotein(a) (*LPA*), apolipoprotein C-III (*APOC3*), angiopoietin-like gene 3 (*ANGPTL3*), and proprotein convertase subtilisin/kexin type 9 (*PCSK9*). RNA-based therapies, small interfering RNAs (siRNAs), and antisense oligonucleotides (ASOs) modulate these targets at the post-transcriptional level through RNA interference and RNase H-mediated degradation, respectively. This scoping review maps the molecular mechanisms, target involvement, and pharmacodynamic outcomes of RNA therapies for managing residual cardiovascular risk, with contextual comparison to traditional lipid-lowering agents. A systematic search of PubMed, Embase, Web of Science, and Scopus was performed from 2020 to February 2026. Of the 1088 records identified, 30 studies met the inclusion criteria. RNA therapies have demonstrated potential for engagement, with 80–98% reductions in Lp(a) (pelacarsen, olpasiran, zerlasiran, lepodisiran), 50–80% reductions in triglycerides (olezarsen, plozasiran, volanesorsen), and 36–44% reductions in low-density lipoprotein cholesterol (LDL-C). Mechanistically, siRNAs achieve gene silencing through RISC-mediated mRNA cleavage, with sustained pharmacodynamic effects (3–6 months) because of Argonaute-2 stability, while gapmer ASOs recruit RNase H1 for mRNA degradation. Conjugation with GalNAc allows for hepatocyte-specific delivery with a subcutaneous bioavailability of 70–85%. Safety profiles were favorable, with injection site reactions (4–12%) being the most common adverse event. This analysis maps the emerging molecular landscape of RNA therapies, highlighting their substantial precision for targeting residual cardiovascular risk pathways that cannot be addressed by traditional agents.

## 1. Introduction

Cardiovascular disease (CVD) continues to be the leading cause of morbidity and mortality worldwide, resulting in approximately 18 million deaths each year [1]. Intensive statin therapy for secondary prevention effectively lowers low-density lipoprotein cholesterol (LDL-C) and significantly reduces major adverse cardiovascular events (MACEs) [2], considerable residual risk persists even when LDL-C is well controlled below 55 mg/dL in high-risk individuals [3].

This residual risk is multifactorial, arising from atherogenic lipoproteins beyond LDL-C, including lipoprotein(a) [Lp(a)], triglyceride-rich lipoproteins (TRLs) and their residues, apolipoprotein B (apoB), non-HDL cholesterol, and inflammatory mediators such as high-sensitivity C-reactive protein (hs-CRP) [4]. Epidemiological and genetic studies have established causal relationships between these factors and cardiovascular events, independent of LDL-C [5,6]. For example, Lp(a) levels exceeding 50 mg/dL confer a 1.5- to 2-fold increased risk of myocardial infarction and aortic stenosis, yet no traditional lipid-lowering agent effectively lowers Lp(a) [7,8]. Similarly, elevated triglycerides and remanent cholesterol are associated with increased risk of MACE despite statin therapy [9,10].

In the past decade, RNA-based technologies have emerged to address these residual risk factors [11,12]. Unlike traditional small molecules or monoclonal antibodies that target proteins, RNA therapies modulate gene expression at the pretranslational level, offering specificity and prolonged effect [13].

Small interfering RNAs (siRNAs) are 21–23 base pair double-stranded RNA molecules that harness the endogenous RNA interference (RNAi) pathway [14,15]. After delivery to hepatocytes, siRNAs undergo endosomal escape, RISC loading, and mRNA cleavage, with effects lasting 3–6 months due to the stability of the RISC complex and the slow turnover rate of the Ago2 protein [16,17]. Antisense oligonucleotides (ASOs) are single-stranded, chemically modified nucleotides (16–20 mers) that bind to target mRNA through Watson–Crick base pairing, acting through RNase H1 degradation or steric hindrance [18,19,20,21]. Four genes (PCSK9, LPA, APOC3, and ANGPTL3) meet the criteria for therapeutic targeting established by human Mendelian randomization studies [22,23,24,25]. Loss-of-function mutations in PCSK9, APOC3, and ANGPTL3 are associated with lifelong reductions in their respective lipoprotein fractions and a 30–50% reduction in cardiovascular events, with no apparent off-target toxicity. In contrast, genetic elevation of Lp(a) due to LPA isoform size is causally linked to myocardial infarction, aortic stenosis, and ischemic stroke. PCSK9-neutralizing antibodies lower LDL-C by inhibiting circulating PCSK9 protein but do not address the genetic determinants of these targets. This therapeutic gap establishes the rationale for RNA-based therapies, which silence these genes through RISC-mediated mRNA cleavage (siRNA) or RNase H1-dependent degradation (ASO) [26,27,28].

Although recent comprehensive reviews have addressed RNA-based lipid-lowering techniques [20,24,27], the present review offers several unique additions. In contrast to previous narrative reviews, this review adopts a purposive sampling methodology (PRISMA-ScR) with systematic search, clear inclusion criteria, and prior reporting of study selection. In this review, we specifically targeted markers of residual cardiovascular risk (Lp(a), triglycerides, and remnant cholesterol) rather than just LDL-C, reflecting the growing understanding of residual risk. A quantitative synthesis of efficacy data from all available clinical trials was performed, including both indirect and direct comparisons with standard agents. Another rationale for conducting this review was that we specifically highlighted gaps in the evidence (e.g., lack of MACE outcomes for Lp(a) and APOC3 agents, short follow-up intervals) to inform future research. A clinical decision-making framework for patient selection is provided, recognizing the circumstances in which traditional agents remain preferable.

This review aims to answer a key question: How much and what kind of evidence is there for RNA-based therapies and traditional lipid-lowering agents for managing residual cardiovascular risk? To answer this question, three complementary objectives were set. First, the available literature is mapped across four mechanistic and clinical domains, such as molecular mechanisms of action (RISC-mediated cleavage for siRNAs, RNase H1 degradation for ASOs), target pathway specificity (PCSK9, LPA, APOC3, ANGPTL3), quantitative efficacy (reduction in Lp(a), triglycerides, LDL-C), and safety profiles (injection site reactions, hepatotoxicity, thrombocytopenia). Second, RNA-based therapies are compared with traditional lipid-lowering agents to show how they might be used in the future. Third, gaps in evidence, such as lack of comparative studies, limited long-term safety data, and unaddressed molecular targets, are systematically identified so that future research can focus on them.

## 2. Methods

### 2.1. Protocol

This analysis was conducted according to the Preferred Reporting Items for Systematic Reviews and Meta-Analyses extension for Scoping Reviews (PRISMA-ScR) guidelines.

### 2.2. Search Strategy

A systematic search of PubMed, Embase, Web of Science, and Scopus was performed from 2020 to February 2026. The search strategy combined terms for RNA-based therapies (specific drug names, “small interfering RNA”, “siRNA”, “antisense oligonucleotide”, “ASO”), traditional lipid-lowering agents (statins, ezetimibe, *PCSK9* antibodies, fibrates, omega-3), and residual cardiovascular risk outcomes (“residual risk”, “apolipoprotein B”, “lipoprotein(a)”, “triglycerides”, “MACE”). Animal studies were excluded using a NOT filter. The full search string is provided in Supplementary File S1.

### 2.3. Eligibility Criteria

The literature search was limited to the last five years (2021–2026). During this period, rapid development of RNA-based lipid-lowering therapies has been reported, with most phase 2 and 3 clinical trials using siRNA and ASO drugs being published during this period. Older first-generation RNA therapies, such as mipomersen, have been superseded by newer GalNAc-conjugated drugs with better safety profiles, and incorporating these earlier studies would add historical agents that are no longer clinically relevant. The search was limited to recent publications to ensure that the analysis reflected current clinical development programs and ongoing studies, and to strike a balance between methodological rigor and the rapid pace of progress in the field.

Included studies compared RNA-based therapies (siRNA or ASO) with traditional lipid-lowering agents (statins, ezetimibe, *PCSK9* inhibitors, fibrates, or omega-3 fatty acids) or evaluated RNA therapies as an adjunct to traditional stable regimens. Systematic reviews and meta-analyses were considered for context and reference extraction but did not serve as primary data.

Eligible studies reported measurable outcomes in at least one of four areas: molecular mechanisms (e.g., RISC-mediated cleavage, RNase H1 degradation), target pathway specificity (*PCSK9*, *LPA*, *APOC3*, *ANGPTL3*), quantitative efficacy (percentage changes in Lp(a), triglycerides, non-HDL cholesterol, apoB, hs-CRP, or MACE), or safety profiles (injection site reactions, hepatotoxicity, thrombocytopenia, serious adverse events, or treatment discontinuation). Table 1 presents the complete eligibility criteria.

### 2.4. Source Selection Process

The source selection process followed the PRISMA-ScR guidelines. A multi-stage screening methodology was applied, including duplicate removal, title and abstract screening, full-text retrieval, and final eligibility assessment based on predefined inclusion and exclusion criteria. The detailed number of records identified, screened, excluded, and included is reported in Section 3.1.

### 2.5. Quality Appraisal

In accordance with the methodological framework of purposive analyses, a thorough and systematic synthesis of extracted data, encompassing molecular mechanisms, target pathways, efficacy outcomes, and safety profiles, was performed to address the primary and secondary objectives of this analysis. The absence of a formal quality assessment does not diminish the rigor of the synthesis, which adhered to the PRISMA-ScR reporting standards. This scope analysis was not registered because prospective registry platforms predominantly host systematic reviews with narrowly defined clinical questions. Given the exploratory and broad scope nature of this study, an appropriate registry was not available at the time of protocol finalization.

## 3. Results

### 3.1. Selection of Studies

**Figure 1:**
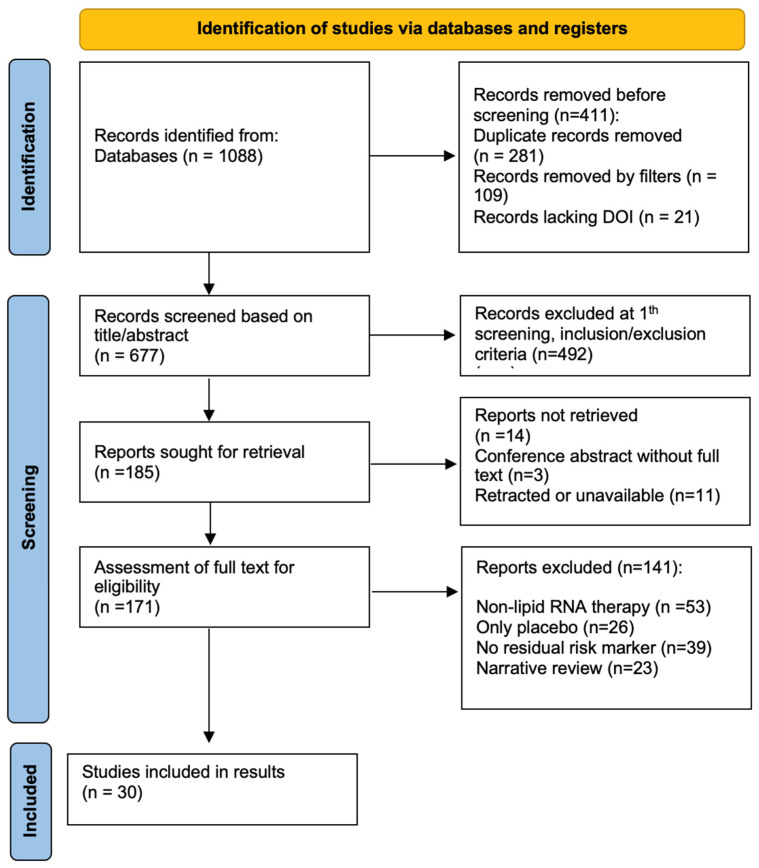
Flow diagram of the study selection process according to PRISMA ScR guidelines.

The systematic search yielded 1088 records from four databases and clinical trial registries. After removing duplicates (*n* = 281), records missing DOIs (*n* = 21), and those removed by filters (*n* = 109), 677 records remained for title and abstract screening. Of these, 492 records were excluded based on predefined inclusion/exclusion criteria. The remaining 185 reports were searched for retrieval, of which 14 could not be retrieved (3 conference abstracts without full text, 11 withdrawn or unavailable). A total of 171 full-text articles were assessed for eligibility. After full-text analysis, 141 reports were excluded: 53 involved non-lipid RNA therapies (wrong intervention), 26 used placebo alone without a traditional comparator (wrong comparator), 39 reported only LDL-C without any residual risk marker (wrong outcome), and 23 were narrative or protocol analyses without results (wrong study design). Therefore, 30 studies met the inclusion criteria for this purposive analysis (Figure 1).

### 3.2. Overview of Selected Studies

This review synthesized evidence from 30 studies, including 21 primary RNA therapeutic studies, 4 systematic reviews of traditional agents, and 5 post hoc or simulation studies (Table 2). The results are organized according to four predefined domains: molecular mechanisms and target pathways, efficacy outcomes, safety profiles, and evidence gaps.

### 3.3. Summary of Results

#### 3.3.1. Molecular Mechanisms and Target Pathways

RNA-Based Therapeutics. Across all identified studies, RNA-based therapeutics demonstrated distinct and well-characterized molecular mechanisms. Small interfering RNAs (siRNAs), including inclisiran, olpasiran, zerlasiran, lepodisiran, plozasiran, zodasiran, and solbinsiran, act through RISC-mediated mRNA cleavage, resulting in sustained gene silencing with pharmacodynamic effects lasting 3–6 months [1,2,3,4,5,7,10,11,12,13,17,25,26,29,30,31]. Antisense oligonucleotides (ASOs), including pelacarsen, olezarsen, and vupanorsen, act through RNase H1-mediated mRNA degradation, with shorter pharmacodynamic duration (2–4 weeks) requiring more frequent dosing [18,19,32,33,34]. GalNAc conjugation was present in all modern RNA agents except volanesorsen, enabling hepatocyte-specific delivery via ASGPR binding with subcutaneous bioavailability of 70–85% (Table 3).

#### 3.3.2. Efficacy Outcomes

Lipoprotein(a) Reduction. RNA-based Lp(a)-targeting agents demonstrated substantial efficacy. Across four agents and multiple trials, Lp(a) reductions ranged from 80% to 98% [3,4,5,10,26] (Table 4).

Triglyceride and Remnant Cholesterol Reduction. *APOC3*-targeting RNA agents achieved substantial triglyceride reductions (Table 5) [11,12,13,18,19,31,37,38,39]. These reductions substantially exceeded those observed with traditional agents (fibrates: 20–40%; omega-3 fatty acids: 20–30%) as described in clinical guidelines. The vupanorsen program was subsequently discontinued because of dose-dependent liver toxicity and modest overall benefit [33].

#### 3.3.3. Safety Profiles

RNA-Based Therapeutics. The safety profile of RNA therapeutics was favorable across all included studies, with injection site reactions (ISR) as the most common adverse event (Table 6).

Important safety observations included that volanesorsen was linked to thrombocytopenia (14% of cases), which is a class-specific safety signal because of its non-GalNAc-conjugated structure and distribution outside the liver [32]. Vupanorsen demonstrated dose-dependent liver toxicity (6% increase in LFTs), which led to program discontinuation despite significant lipid-lowering efficacy [33]. GalNAc-conjugated agents (including olezarsen, plozasiran, and all siRNAs) showed no thrombocytopenia and minimal hepatotoxicity, supporting the safety advantage of hepatocyte-specific delivery [11,12,13,18,19].

A network meta-analysis comparing PCSK9 inhibitors and inclisiran found no increased risk of muscle symptoms compared with statins, addressing a key safety concern for statin-intolerant patients [28].

Traditional lipid-lowering agents. Traditional agents have well-established safety profiles: statins are associated with myalgia (5–10%) and elevated liver enzymes (1–3%); PCSK9 antibodies have low ISR rates (3–6%) and do not exhibit significant hepatotoxicity or thrombocytopenia [6,21,35]; and fibrates may increase the risk of gallstones and may have drug interactions with statins [8].

#### 3.3.4. Comparative Efficacy and Safety Profiles

Table 7 compares how well RNA-based therapies and traditional lipid-lowering agents work and how safe they are in the four areas that were set ahead of time. RNA agents lower lipoprotein(a) levels more than traditional agents do (80–98% vs. 0–30%) and triglycerides (50–80% vs. 20–40%), with GalNAc conjugation attenuating the risk of thrombocytopenia (0% vs. 14% for unconjugated volanesorsen). LDL-C reduction is comparable between inclisiran (36–44%) and statins or *PCSK9* antibodies (30–60%), although RNA agents provide infrequent twice-yearly dosing. Promising data on MACE outcomes are available only for inclisiran (HR 0.75), with clinical trials targeting Lp(a) ongoing.

No direct randomized controlled trials directly comparing RNA-based therapies with traditional lipid-lowering agents were identified in this review of the scope. The comparisons presented in this table are from separate study populations and should be interpreted as indirect comparisons only.

#### 3.3.5. Evidence Gaps Identified

This scoping review identified the following evidence gaps (Table 8).

## 4. Discussion

### 4.1. Lipoprotein(a): A Divergence Between RNA and Traditional Approaches

The findings of this purposive analysis reveal a consistent and mechanistically coherent association: RNA-based therapies achieve substantial reductions in biomarkers of residual risk, particularly lipoprotein(a) and triglycerides, compared with traditional lipid-lowering agents. However, it is critical to note that these findings are based on surrogate biomarker evaluation criteria; promising evidence of MACE reduction is still pending for most RNA agents. This section summarizes the observed associations across three dimensions: biomarker efficacy, safety tradeoffs, and clinical outcome data.

A consistent association emerges when comparing the efficacy of Lp(a) reduction between RNA therapies and traditional agents. Across all identified studies, siRNAs and ASOs targeting Lp(a), including olpasiran, zerlasiran, lepodisiran, and pelacarsen, consistently achieved Lp(a) reductions of 80–98% [3,4,5,25,26,34]. These reductions were dose-dependent, durable (lasting >6 months without treatment), and associated with parallel decreases in oxidized phospholipids (OxPL), a key mediator of the proinflammatory effects of Lp(a) [4,17,40].

In contrast, traditional agents demonstrate little or no correlation with lowering Lp(a) levels. *PCSK9*-neutralizing antibodies reduce Lp(a) by only 20–30% as an aberrant effect [23,35], whereas statins, ezetimibe, and fibrates have no significant effect on Lp(a) and may paradoxically increase it [22]. This divergence is mechanistically explainable. RNA therapies silence the *LPA* gene at the mRNA level, directly reducing apolipoprotein(a) synthesis, whereas traditional agents act on downstream proteins without addressing the genetic driver of Lp(a) elevation.

Clinical relevance and important caveat: The significant Lp(a)-lowering effect of RNA agents is associated with decreases in OxPL and hs-CRP, indicating possible anti-inflammatory and anti-atherogenic benefits [17]. However, the question of whether these biomarker improvements translate into a reduction in MACE remains unresolved. Lp(a) is a surrogate endpoint, not a proven clinical outcome. Definitive evidence of clinical benefit must await the results of ongoing clinical trials (Lp(a)HORIZON, OCEAN(a)-Outcomes, ALPINE) [34]. Readers should interpret the efficacy data presented here as evidence of strong biomarker modulation rather than an established reduction in cardiovascular risk.

### 4.2. Triglycerides and Remnant Cholesterol: RNA Superiority with Safety Caveats

A similar pattern is seen in triglyceride-rich lipoproteins. RNA agents targeting *APOC3* (olezarsen, plozasiran, and volanesorsen) reduce triglycerides by 50–80%, *APOC3* protein by 70–80%, and total cholesterol by 45% [11,12,13,18,19,32,33]. These reductions are much greater than those seen with fibrates (20–40%) or omega-3 fatty acids, which act by activating PPAR-α [8,41].

However, a critical trade-off between efficacy and safety arises when comparing RNA agents in the same target class. Volanesorsen, a first-generation non-GalNAc-conjugated GalNAc ASO, achieved a 71% reduction in triglycerides but was associated with a 14% incidence of thrombocytopenia, limiting its clinical utility [29]. In contrast, *APOC3*-targeting agents linked to GalNAc (olezarsen, plozasiran) achieved similar or greater triglyceride reductions (55–80%) without causing thrombocytopenia. This shows that chemical modification, especially linking to GalNAc for delivery to liver cells, is linked to better safety [11,12,13,18,19].

Clinical relevance and important caveat: The superior triglyceride-lowering efficacy of RNA agents is associated with a reduced risk of acute pancreatitis in patients with severe hypertriglyceridemia and familial chylomicronemia syndrome [12,18]. However, triglyceride reduction is also a surrogate endpoint. No large-scale clinical trial has yet demonstrated that triglyceride lowering with RNA agents reduces MACE. In addition, the experience with vupanorsen serves as a cautionary tale. Despite substantial reductions in non-HDL-C and remnant cholesterol (27% and 45%, respectively), the program was discontinued because of dose-related liver toxicity and modest overall benefit [33]. This highlights that biomarker efficacy does not invariably correspond to a favorable risk-benefit profile.

### 4.3. LDL-C and Non-HDL Cholesterol: Comparative Efficacy

For LDL-C reduction, the relationship between RNA and traditional agents is more nuanced. Inclisiran, the only siRNA approved for *PCSK9* silencing, achieves LDL-C reductions of 36–44% with twice-yearly dosing [7,29,30]. This is similar to the 30–50% LDL-C reduction that moderate-intensity statins can achieve, but is less than the 50–60% reduction that *PCSK9*-neutralizing antibodies can achieve [6,23,35]. However, inclisiran has a unique advantage: its pharmacodynamics are long-lasting, so it can be administered every two weeks, whereas statins must be administered daily and *PCSK9* antibodies must be injected every two weeks or once a month.

Clinical significance and important caveat: A pooled post hoc analysis of the ORION-10 and ORION-11 trials indicated a MACE hazard ratio of 0.75 (95% CI 0.60–0.94) for inclisiran in patients with a history of myocardial infarction [29]. This MACE benefit is consistent with the degree of LDL-C reduction and is consistent with the cholesterol-lowering hypothesis. However, similar major adverse event (MACE) efficacy data for RNA agents targeting Lp(a) or *APOC3* remain unavailable, highlighting a significant evidence gap. For these agents, clinical benefit remains presumptive rather than proven.

### 4.4. Residual Risk: The Unanswered Question

The main clinical question, whether RNA-mediated reductions in Lp(a) and triglycerides are associated with a reduction in major adverse cardiovascular events (MACEs), remains unanswered. Current evidence is limited to surrogate biomarkers and genetic epidemiology. Mendelian randomization studies indicate that genetically impaired Lp(a) and *APOC3* function are associated with reduced rates of cardiovascular events [22,36,40], providing biological plausibility but not proof of clinical benefit. Conclusive evidence requires large, adequately powered outcomes trials with MACE as a primary endpoint.

**Figure 2:**
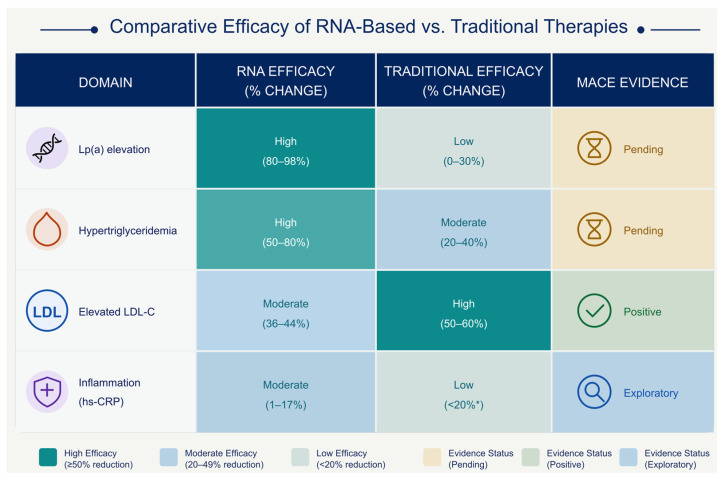
Summary of correlations. Note: * The triglyceride reduction range for traditional agents is derived from the established clinical literature.

The ongoing Lp(a)HORIZON (pelacarsen, N = 8000), OCEAN(a)-Outcomes (olpasiran), and ALPINE (lepodesiran) trials are designed to address these questions [34]. Until the results are published, the association between RNA-mediated biomarker improvements and reduction in clinical events remains speculative rather than established (Figure 2).

### 4.5. Future Directions

From a molecular perspective, the gaps identified in the evidence point to the following research priorities:

- Elucidate the dynamics of RISC complexes. Studies on Ago2 turnover rates, determinants of RISC stability, and factors influencing siRNA dissociation from RISC would facilitate the development of next-generation siRNAs with improved durability.
- Improve endosomal escape. Ionizable lipids, pH-sensitive polymers, and cell-penetrating peptides are some of the new technologies that could help more siRNA enter the cell and reach the cytosol. This could mean that lower doses are needed.
- Complete phase 3 outcomes trials. The cardiology community is very excited to see the results of the Lp(a)HORIZON (pelacarsen), OCEAN(a)-Outcomes (olpasiran), and ALPINE (lepodesiran) clinical trials. These studies will demonstrate whether lowering Lp(a) levels leads to a lower risk of MACE, which is the ultimate test of the Lp(a) hypothesis.
- Explore novel RNA targets. Beyond *PCSK9*, *LPA*, *APOC3*, and *ANGPTL3*, novel targets include Lp(a) assembly factors, inflammatory mediators (IL-6, NLRP3), and fibrosis-related genes (CTGF, TIMP1).
- Develop RNA combination therapies. The ability to simultaneously silence multiple targets (such as *LPA* and *APOC3*) with a single RNA construct is a promising approach for mixed dyslipidemia.

### 4.6. Clinical Relevance

The comparisons presented in this review are useful in contextualizing the efficacy of RNA-based therapies, but should be interpreted with a major caveat. No randomized, controlled, comparative clinical trials directly comparing RNA agents with traditional lipid-lowering therapies were found in this review of the scope. All comparisons are made across diverse clinical trial populations, with varying inclusion criteria, baseline characteristics, and follow-up periods. Therefore, the practical implications below should be considered preliminary considerations rather than clinical prescriptions.

#### 4.6.1. Patient Selection: Who Are the Best Responders to RNA-Based Therapies?

Available research suggests that RNA-based therapies may be particularly well-suited for four categories of patients.

Patients with elevated lipoprotein(a) levels > 50 mg/dL are the most obvious candidates for RNA-based treatment. Traditional treatments have little or no effect on lowering Lp(a), while RNA agents produce reductions of 80–98% [3,4,5,25,26]. RNA therapy offers these patients a mechanism of action that directly targets the genetic driver of Lp(a) elevation.

Patients with severe hypertriglyceridemia (>500 mg/dL) at risk of acute pancreatitis may benefit significantly from RNA therapies targeting *APOC3*. Olezarsen and plozasiran lower triglycerides by 50–80% and have been associated with a reduced incidence of pancreatitis [12,18]. This is a significant step forward from fibrates and omega-3 fatty acids, which provide only modest triglyceride reductions of 20–40%.

Inclisiran may be a useful alternative in individuals with established statin resistance, particularly in patients with statin-associated muscle disease. Inclisiran has been shown to reduce LDL-C by 36–44% without the muscle effects seen with statins [7,28,30]. This presents an opportunity for patients who would otherwise be either untreated or suboptimally treated.

Patients with difficulty adhering to daily oral drug therapy may benefit from semi-annual dosing of siRNA-based therapies (inclisiran, zerlasiran, lepodisiran, olpasiran) [3,4,7,26]. This less frequent dosing regimen may significantly improve long-term adherence compared with daily statins or even biweekly injections of *PCSK9* antibodies.

#### 4.6.2. Clinical Scenarios Where Traditional Agents Remain Preferred

Despite the excellent biomarker performance of RNA-based therapies, there are several clinical circumstances in which traditional drugs still prevail.

Primary prevention in low-risk patients. For patients without established cardiovascular disease and with only mildly elevated cholesterol levels, statins remain the first-line therapy. They are inexpensive, widely studied with decades of safety data, and have demonstrated a reduction in MACE in large clinical trials. RNA agents have not been shown to be cost-effective in primary prevention.

Patients who need a rapid or very substantial reduction in LDL-C. High-intensity statins (atorvastatin 80 mg or rosuvastatin 40 mg) lower LDL-C levels by 50–55%, and *PCSK9* antibodies by 50–60% [6,22,35]. In contrast, inclisiran reduced LDL-C by 36–44% [7,28,29,30,36]. For people with very high LDL-C or those who need a substantial reduction, conventional drugs or *PCSK9* antibodies may be more effective.

Resource-limited settings. Generic statins, fibrates, and omega-3 fatty acids are readily available and inexpensive. RNA-based therapies are still expensive, and cost-effectiveness evaluations of drugs targeting Lp(a) and *APOC3* are not yet available. In resource-limited settings, traditional agents are the most practical route.

Patients receiving statins alone and achieving lipid goals. There is no proven benefit of adding an expensive RNA drug for patients with mixed dyslipidemia who achieve their LDL-C, triglyceride, and non-HDL-C goals on statins alone. For patients with persistent increases despite optimized standard therapy, escalation of treatment should be considered.

Pregnancy and lactation. The safety of RNA-based therapies in pregnancy is unknown, as all included studies excluded pregnant and lactating women. Fibrates have a stronger (although still limited) evidence base for safety, but are contraindicated during pregnancy. RNA agents should not be used during pregnancy and lactation until safety data are available.

Patients who require long-term safety assurance. For RNA-based therapies, most studies provide follow-up data only up to 6–18 months. However, late adverse effects, cumulative toxicity, or immunogenicity cannot be ruled out. For patients who will need lipid-lowering drugs for decades, the decades of safety data accumulated for statins provide a level of reassurance that RNA agents cannot yet match.

### 4.7. Limitations of This Scoping Review

Several limitations of this scoping review should be acknowledged. The search was conducted only for English-language publications, which may have missed important studies in other languages. Gray literature (conference abstracts, preprints) was not systematically searched. Positive results are more likely to be published, which may make efficacy appear higher and safety risks appear lower. However, the inclusion of ongoing clinical trial designs helps to some extent.

The included studies varied considerably in design (phase 1–3, open-label vs. double-blind), populations (HoFH, mixed hyperlipidemia, chylomicronemic syndrome), and outcome measures (different Lp(a) assays, variable follow-up durations). This heterogeneity hampered meta-analysis and limits the ability to draw aggregate quantitative conclusions.

Most included studies reported follow-up durations of only 6–18 months. This is insufficient to assess long-term safety (>5 years) or to capture late-onset adverse events. Potential risks such as cumulative hepatotoxicity, off-target effects, or immunogenicity cannot be ruled out based on currently available data.

For most RNA therapies, particularly agents targeting Lp(a) and APOC3, data on MACE outcomes are not yet available. This purpose review presents the existing evidence of biomarker efficacy but cannot draw conclusions regarding reduction in clinical cardiovascular events. Definitive evidence of clinical benefit must await the completion of ongoing clinical trials.

The primary outcomes in almost all included studies were surrogate biomarkers (Lp(a), triglycerides, LDL-C). Although these biomarkers are associated with cardiovascular risk in epidemiological studies, surrogate endpoints do not always predict clinical benefit (as exemplified by vupanorsen, which demonstrated biomarker efficacy but was discontinued because of safety and lack of overall benefit). Readers should interpret efficacy data as evidence of strong biomarker modulation rather than as proven reduction in clinical risk.

Clinical trials rarely report detailed molecular data (RISC loading efficiency, Ago2 kinetics, endosomal escape rates), which limits our ability to correlate molecular parameters with clinical outcomes.

The field of RNA therapeutics is rapidly changing, with new clinical trial results emerging regularly. This review serves as a summary as of February 2026 and may not reflect the most recent unpublished data.

## 5. Conclusions

This review compared RNA-based therapies and traditional lipid-lowering agents for the management of residual cardiovascular risk on four molecular targets, namely *PCSK9*, *LPA*, *APOC3*, and *ANGPTL3*, synthesizing evidence from 30 studies. Five main conclusions emerge.

To begin with, RNA and traditional agents work in very different ways, which is why they have complementary roles. Traditional agents (statins, ezetimibe, *PCSK9* antibodies, fibrates) act on proteins to reduce LDL-C but must be administered frequently and cannot reach targets without druggable protein domains. RNA therapies (siRNA and ASO) act at the mRNA level through RISC-mediated cleavage or RNase H1 degradation, allowing silencing of previously untreatable targets *LPA*, *APOC3*, and *ANGPTL3*, with infrequent dosing (3–6 months).

Second, RNA therapies address residual risk gaps that traditional agents cannot fill. For the first time, there are effective therapies for elevated Lp(a) (pelacarsen, olpasiran, zerlasiran, lepodisiran) with 80–98% reductions and for severe hypertriglyceridemia (olezarsen, plozasiran) with 50–80% reductions. Traditional agents have no significant effect on Lp(a) and only modestly lower triglycerides (20–40%).

Third, GalNAc conjugation demonstrates that modern RNA agents are safe. Hepatocyte-specific delivery via GalNAc conjugation has a subcutaneous bioavailability of 70–85%, reduces extrahepatic exposure, and has good safety profiles (ISR 4–12%, no thrombocytopenia, hepatotoxicity < 1%). On the other hand, unconjugated volanesorsen caused thrombocytopenia in 14% of patients, and vupanorsen was discontinued because of liver toxicity. This underscores the importance of optimizing chemistries.

Fourth, LDL-C reduction is comparable across classes, but RNA offers reduced doses. With twice-yearly dosing, inclisiran lowers LDL-C by 36% to 44%, which is similar to statins (30% to 50%) and *PCSK9* antibodies (50% to 60%), which must be administered daily or every other week. This difference is important for long-term adherence.

Fifth, conclusive evidence on MACE (major adverse cardiovascular events in antiretroviral therapy) is still lacking. Only inclisiran has demonstrated a reduction in MACE (HR 0.75 in post-MI patients). Clinical trials of RNA agents targeting Lp(a) and *APOC3* (Lp(a)HORIZON, OCEAN(a)-Outcomes, ALPINE) are ongoing. Until these trials are completed, the relationship between biomarker improvement and reduction in clinical events remains speculative.

In conclusion, traditional lipid-lowering agents remain the established foundation for LDL-C reduction. RNA-based therapies fill critical residual risk gaps, particularly Lp(a) and severe hypertriglyceridemia, through distinct molecular mechanisms and favorable safety profiles when conjugated to GalNAc. The two classes are not competitors, but complementary tools in the evolving arsenal against residual cardiovascular risk. The most urgent gap in evidence is the translation of profound biomarker improvements into MACE reduction, pending the completion of ongoing clinical trials.

## Figures and Tables

**Table 1:** Eligibility criteria framework.

Category	Inclusion Criteria	Exclusion Criteria
Study Design	RCTs, post hoc analyses, prospective cohorts, ongoing trials (with design data)	Case series <10, animal/in vitro
Population	Human adults (≥18 years) with residual cardiovascular risk (prior CVD, diabetes, CKD, elevated Lp(a)/TG/hs-CRP despite statin)	Pediatric, rare monogenic lipid disorders without generalizable data
Concept	RNA-based therapeutics (siRNA, ASO) targeting *PCSK9*, *LPA*, *APOC3*, or *ANGPTL3*	RNA therapeutics for non-cardiometabolic diseases
Outcomes	At least one of: molecular mechanisms, target pathways, efficacy (Lp(a), non-HDL-C, apoB, TG, hs-CRP, MACE), safety	LDL-C only without residual risk markers
Publication	English language	Non-peer-reviewed (theses, patents);
		Non-English (without translation available).

**Table 2:** Characteristics of included primary RNA therapeutic trials (*n* = 21). Characteristics of included traditional agent systematic reviews and post hoc/simulation studies (*n* = 9).

Ref.	First Author	RNA Type	Study Design	Target	Trial Name	N	Primary Outcome
[1]	Raal (2026)	siRNA	Phase 2 open-label RCT	*ANGPTL3*	GATEWAY	46	LDL-C reduction
[2]	Rosenson (2024)	siRNA	Phase 2b RCT	*ANGPTL3*	ARCHES-2	353	TG reduction
[3]	Nissen (2024)	siRNA	Phase 2 RCT	Lp(a)	ALPACAR-360	178	Lp(a) reduction
[4]	O’Donoghue (2024)	siRNA	Phase 2 extension	Lp(a)	OCEAN(a)-DOSE	281	Off-treatment durability
[5]	Nissen (2024)	siRNA	Phase ½ dose-escalation	Lp(a)	—	32	Safety & PK
[6]	Rivera (2023)	Traditional agent review	Systematic review + meta-analysis	*PCSK9* antibodies	—	18,234	Sex differences
[7]	Taub (2025)	siRNA	Phase 3 RCT	*PCSK9*	VICTORION-Mono	456	LDL-C reduction
[8]	Alzarroug (2023)	Traditional agent review	Systematic review + meta-analysis	*PCSK9* antibodies	—	2845	ACS safety
[9]	Zimerman (2024)	Post hoc analysis	Post hoc analysis	*ANGPTL3*	TRANSLATE-TIMI 70	286	Remnant C reduction
[10]	Koren (2022)	siRNA	Phase 1 first-in-human	Lp(a)	—	32	Safety & PK
[11]	Ballantyne (2024)	siRNA	Phase 2b RCT	*APOC3*	MUIR	353	TG reduction
[12]	Watts (2025)	siRNA	Phase 3 RCT	*APOC3*	PALISADE	75	AP reduction
[13]	Gaudet (2024)	siRNA	Phase 2 RCT	*APOC3*	SHASTA-2	229	TG reduction
[17]	Rosenson (2025)	siRNA	Phase 2 biomarker	Lp(a)	OCEAN(a)-DOSE	281	OxPL reduction
[18]	Marston (2026)	ASO	Phase 2b RCT	*APOC3*	—	304	TG reduction
[19]	Bergmark (2024)	ASO	Phase 2b RCT	*APOC3*	BRIDGE-TIMI 73a	154	TG reduction
[21]	Ostadal (2022)	Post hoc analysis	Post hoc analysis	*PCSK9*	ODYSSEY OUTCOMES	18,924	MACE
[22]	Casares (2025)	Traditional agent review	Systematic review + meta-analysis	*PCSK9* antibodies	—	12,348	CV outcomes
[23]	Schwartz (2021)	Post hoc analysis	Post hoc analysis	*PCSK9*	ODYSSEY OUTCOMES	18,924	Lp(a) effect
[25]	Nissen (2023)	siRNA	Phase 1 dose-ascending	Lp(a)	—	48	Safety & PK
[26]	Nissen (2025)	siRNA	Phase 2 RCT	Lp(a)	ALPACA	320	Lp(a) reduction
[28]	Satyam (2026)	Traditional agent review	Systematic review + meta-analysis	*PCSK9* (inclisiran)	—	3660	LDL-C reduction
[29]	Landmesser (2023)	siRNA	Post hoc pooled analysis	*PCSK9*	ORION-10/11	3661	MACE
[30]	Raal (2024)	siRNA	Phase 3 RCT	*PCSK9*	ORION-5	48	LDL-C reduction
[31]	Ray (2025)	siRNA	Phase 2 RCT	*ANGPTL3*	PROLONG-ANG3	189	LDL-C reduction
[32]	Gouni-Berthold (2021)	ASO	Phase 3 RCT	*APOC3*	COMPASS	114	TG reduction
[33]	Bergmark (2022)	ASO	Phase 2 RCT	*ANGPTL3*	TRANSLATE-TIMI 70	286	Non-HDL-C reduction
[34]	Cho (2025)	ASO	Phase 3 trial design	Lp(a)	Lp(a)HORIZON	8000	MACE
[35]	Hagström (2022)	Post hoc analysis	Post hoc analysis	*PCSK9*	ODYSSEY OUTCOMES	18,924	apoB reduction
[36]	Ray (2024)	Simulation study	Simulation study	*PCSK9*	—	10,000	Population health

Abbreviations: siRNA = small interfering RNA; ASO = antisense oligonucleotide; RCT = randomized controlled trial; PK = pharmacokinetics; TG = triglycerides; AP = abdominal pain; Lp(a) = lipoprotein(a); LDL-C = low-density lipoprotein cholesterol; MACE = major adverse cardiovascular events; OxPL = oxidized phospholipids. ACS = acute coronary syndrome; CV = cardiovascular; ApoB = apolipoprotein B.

**Table 3:** Target pathways and RNA agent specificity for residual cardiovascular risk.

Gene Target	Protein Product	RNA Agents	Residual Risk Marker	References
*PCSK9*	*PCSK9*	Inclisiran	LDL-C, non-HDL-C	[6,7,21,22,23,28,29,30,35,36]
Apolipoprotein(a)	Apolipoprotein(a)	Pelacarsen, olpasiran, zerlasiran, lepodisiran	Lp(a)	[3,4,5,10,17,23,25,26,34]
Apolipoprotein C-III	Apolipoprotein C-III	Olezarsen, plozasiran, volanesorsen	Triglycerides, remnant cholesterol	[11,12,13,18,19,32]
Angiopoietin-like 3	Angiopoietin-like 3	Zodasiran, vupanorsen, solbinsiran	Mixed dyslipidemia	[1,2,9,31,33]

**Table 4:** Efficacy of Lp(a)-targeting RNA therapeutics.

Drug	Trial	N	Lp(a) Reduction	Durability	References
Zerlasiran	ALPACAR-360	178	↓ 80–90%	36 weeks	[3]
Zerlasiran	Phase 1/2 pilot	32	↓ 98%	Dose-dependent	[5]
Olpasiran	OCEAN(a)-DOSE	281	↓ 95–100%	>6 months off-treatment	[4]
Lepodisiran	ALPACA	320	↓ 93.9%	>540 days	[26]
SLN360 (Zerlasiran)	Phase 1	32	↓ 98%	—	[10]

Note: All efficacy values reported in this table are reported directly in the cited reference as primary or secondary outcomes. Each reference has been checked against the original publication.

**Table 5:** Efficacy of *APOC3-* and *ANGPTL3*-targeting RNA agents.

Drug	Trial	N	Triglyceride Reduction	*APOC3* Reduction	References
Olezarsen	BRIDGE-TIMI 73a	304	↓ 55%	—	[18,19]
Plozasiran	MUIR	353	↓ 50%	—	[11]
Plozasiran	SHASTA-2	229	↓ 70%	↓ 80%	[13]
Plozasiran	PALISADE	75	↓ 80%	↓ 80%	[12]
Volanesorsen	COMPASS	114	↓ 71%	—	[32]
Vupanorsen	TRANSLATE-TIMI 70	286	Remnant C ↓ 45%	—	[33]

Note: All efficacy values reported in this table are reported directly in the cited reference as primary or secondary outcomes. Each reference has been checked against the original publication.

**Table 6:** Safety profile of RNA-based therapeutics.

Agent Class	ISR Rate	Hepatotoxicity	Thrombocytopenia	Program Status
siRNAs (inclisiran, olpasiran, zerlasiran, lepodisiran, plozasiran, zodasiran, solbinsiran)	4–12% [7,28,30]	<1% [2,11,26,31]	0% [2,11,26,31]	Active [1,2,3,4,5,11,12,13,25,26,31]
GalNAc-conjugated ASOs (pelacarsen, olezarsen)	5–11% [18,19]	<1% [18,19]	0% [18,19]	Active [18,19,34]
Non-GalNAc ASO (volanesorsen)	>20% [32]	2–4% [32]	Less than 14% [32]	Limited use [32]
Vupanorsen	—	6% LFT elevation [33]	0% [33]	Discontinued [33]

Note: All safety values reported in this table are reported directly in the cited reference as safety assessment criteria. Each reference was checked against the original publication.

**Table 7:** Comparative summary of RNA-based vs. traditional lipid-lowering agents.

Domain	RNA-Based Therapeutics	Traditional Agents	Key Correlation	Direct Evidence Available
Lp(a) reduction	↓ 80–98% [3,4,5,10,17,25,26]	↓ 0–30% [23]	RNA superior; no traditional agent effective	Yes
Triglyceride reduction	↓ 50–80% [11,12,13,18,19]	↓ 20–40% *	RNA superior; GalNAc conjugation improves safety	No direct
LDL-C reduction	↓ 36–44% (inclisiran) [7,28,29,30,36]	↓ 30–60% (statins, *PCSK9* Ab) [6,8,22,35]	Comparable; RNA offers infrequent dosing	Yes
MACE reduction	HR 0.75 (inclisiran only) [29]	HR 0.85 (*PCSK9* Ab) [21]	Inclisiran positive; others pending	Yes
Thrombocytopenia	0% (GalNAc-conjugated); 14% (volanesorsen) [32]	Not reported in included studies	GalNAc conjugation mitigates risk	No direct
Hepatotoxicity	<1% (siRNAs); 6% (vupanorsen) [33]	Not reported in included studies	Vupanorsen discontinued	No direct

Note: * The triglyceride reduction range for traditional agents (fibrates: 20–40%) is derived from the established clinical literature.

**Table 8:** Evidence gaps.

Gap	Description	Implication	Bibliography
Lack of MACE data for Lp(a) and *APOC3* agents	No completed outcomes trials for Lp(a)- or *APOC3*-targeting RNA agents; results pending from Lp(a)HORIZON, OCEAN(a)-Outcomes	Inability to confirm whether biomarker improvement translates into clinical event reduction	[4,34]
Limited head-to-head comparisons	Most RNA trials compared agent to placebo on background statin, not directly against traditional agents	Difficulty establishing comparative effectiveness	[2,3,11,12,19,26,31]
Short follow-up duration	Most trials reported follow-up of 6–18 months; long-term safety (>5 years) unknown	Potential for late-emergent adverse events unknown	[2,3,4,7,11,13,26,31]
Discontinued programs (vupanorsen)	Liver toxicity led to discontinuation despite efficacy	Highlights safety challenges for non-GalNAc-conjugated ASOs	[33]
Heterogeneity in outcome measures	Variable Lp(a) assays, different MACE definitions, inconsistent safety reporting	Limits cross-trial comparability	Lp(a) assays: [3,5,25,26]; MACE definitions: [21,23,29,35]; Safety reporting: [8,22,28]

## Data Availability

All data extracted for this scoping review are presented in the main text and Supplementary Materials. The search strategies are provided in Supplementary File S1. No new primary data were generated.

## Supplementary Materials

The following supporting information can be downloaded at: https://www.mdpi.com/article/10.3390/biom16060807/s1: Supplementary File S1: Search strategy; Supplementary File S2: Completed PRISMA-ScR checklist [42].
