# Supplementary material for: Molecular Mechanisms and Therapeutic Targets of RNA-Based and Traditional Lipid-Lowering Agents in Residual Cardiovascular Risk: A Scoping Review of Key Directions Towards Future Perspectives
Source: Biomolecules. 2026 May 29;16(6):807. doi: 10.3390/biom16060807 (PMC13296526; doi:10.3390/biom16060807)
Supplement: Supplementary file 1 [file biomolecules-16-00807-s001.zip › Supplementary File S1.pdf]

Supplementary File S1
Keywords and Subject Headings Used During the Search

Database	Search strategy
PubMed accessed February, 2026	("RNA, Small Interfering"OR "Oligonucleotides, Antisense" OR "Angiopoietin-Like Proteins"OR "Lipoprotein(a)"OR "PCSK9 Inhibitors"OR "Lipidomics")<br>AND<br>("antisense oligonucleotide"OR "small interfering RNA"OR "RNA interference"OR "lipoprotein(a)"OR "Lp(a) " OR "apolipoprotein C-III"OR apoC-III OR ANGPTL3 OR "angiopoietin-like 3"OR "PCSK9")<br>AND<br>("Cardiovascular Diseases" OR "Atherosclerosis" OR "Heart Disease Risk Factors" OR "Residual Risk")<br>NOT<br>("Animals" OR "Cell Line"OR "In Vitro Techniques"OR "Rats"OR "Mice")
Scopus accessed February, 2026	("RNA-based therapeutics" OR "small interfering RNA" OR "antisense oligonucleotide" OR "aso" OR "inclisiran"OR "pelacarsen" OR "olezarsen" OR "volanesorsen")<br>AND<br>("lipoprotein(a)" OR "Lp(a)" OR pcsk9 OR angptl3 OR "apolipoprotein C-III" OR "apoC-III" OR "triglyceride-rich lipoproteins") OR (statin OR statins OR ezetimibe OR "PCSK9 monoclonal antibody" OR alirocumab OR evolocumab OR "traditional lipid-lowering agents")<br>AND ("residual cardiovascular risk" OR "residual risk")<br>AND NOT<br>("Animals" OR "Cell Line" OR "In Vitro Techniques")<br><br>AND PUBYEAR > 2020 AND PUBYEAR < 2026
Web Of Science accessed February, 2026	TS=("lipoprotein(a)" OR "Lp(a)" OR "remnant cholesterol" OR "triglyceride-rich lipoprotein*" OR "apolipoprotein B" OR "apoB" OR "apoC-III" OR "apolipoprotein C-III" OR "ANGPTL3" OR "angiopoietin-like 3" OR "PCSK9" OR "inclisiran" OR "pelacarsen" OR "solbinsiran" OR "plozasiran" OR "small interfering RNA" OR "siRNA" OR "antisense oligonucleotide*" OR "ASO" OR "lipidomic")<br>AND<br>("cardiovascular risk" OR "residual risk")<br>AND<br>NOT TS=("animal model" OR "in vitro" OR "cell line" OR "mouse" OR "rat")
Embase accessed February, 2026	exp RNA interference/ OR exp small interfering RNA/ OR exp antisense oligonucleotide/ OR (inclisiran OR pelacarsen OR olpasiran OR zerlasiran OR lepodisiran OR olezarsen OR plozasiran OR zodasiran OR solbinsiran OR vupanorsen OR volanesorsen).<br>AND<br>exp statin/ OR exp hydroxymethylglutaryl-coenzyme A reductase inhibitor/ OR<br>exp ezetimibe/ OR exp PCSK9 inhibitor/ OR exp proprotein convertase 9 antibody/ OR<br>AND

	exp major adverse cardiac event/ OR exp cardiovascular mortality/ OR r esidual cardiovascular risk OR residual risk OR non-HDL cholesterol OR n on-HDL-C<br>AND<br>apolipoprotein B OR apoB OR lipoprotein(a) OR Lp(a) OR triglycerides O R TG OR hs-CRP OR high-sensitivity C-reactive protein OR MACE OR ma jor adverse cardiovascular events
